# Building transformers from neurons and astrocytes

**DOI:** 10.1073/pnas.2219150120

**Published:** 2023-08-14

**Authors:** Leo Kozachkov, Ksenia V. Kastanenka, Dmitry Krotov

**Affiliations:** ^a^Massachusetts Institute of Technology-International Business Machines, Watson Artificial Intelligence Laboratory, IBM Research, Cambridge, MA 02142; ^b^Department of Brain and Cognitive Sciences, Massachusetts Institute of Technology, Cambridge, MA 02139; ^c^Department of Neurology, MassGeneral Institute for Neurodegenerative Diseases, Massachusetts General Hospital and Harvard Medical School, Boston, MA 02115

**Keywords:** neuroscience, astrocytes, Transformers, glia, artificial intelligence

## Abstract

Glial cells account for between 50% and 90% of all human brain cells, and serve a variety of important developmental, structural, and metabolic functions. Recent experimental efforts suggest that astrocytes, a type of glial cell, are also directly involved in core cognitive processes such as learning and memory. While it is well established that astrocytes and neurons are connected to one another in feedback loops across many timescales and spatial scales, there is a gap in understanding the computational role of neuron–astrocyte interactions. To help bridge this gap, we draw on recent advances in AI and astrocyte imaging technology. In particular, we show that neuron–astrocyte networks can naturally perform the core computation of a Transformer, a particularly successful type of AI architecture. In doing so, we provide a concrete, normative, and experimentally testable account of neuron–astrocyte communication. Because Transformers are so successful across a wide variety of task domains, such as language, vision, and audition, our analysis may help explain the ubiquity, flexibility, and power of the brain’s neuron–astrocyte networks.

## Significance

Transformers have become the default choice of neural architecture for many machine learning applications. Their success across multiple domains such as language, vision, and speech raises the question: How can one build Transformers using biological computational units? At the same time, in the glial community, there is gradually accumulating evidence that astrocytes, formerly believed to be passive house-keeping cells in the brain, in fact play an important role in the brain’s information processing and computation. In this work we hypothesize that neuron–astrocyte networks can naturally implement the core computation performed by the Transformer block in AI. The omnipresence of astrocytes in almost any brain area may explain the success of Transformers across a diverse set of information domains and computational tasks.

Astrocytes, one kind of glia, are a ubiquitous cell type in the central nervous system. It is empirically well established that astrocytes and neurons communicate with one another via feedback loops that span many spatial and temporal scales (1–3). These communications underlie a variety of important physiological processes, such as regulating blood flow to neurons (4) and eliminating debris (5). A rapidly growing body of evidence suggests that astrocytes also play an active and flexible role in behavior (6–12). However, a firm computational interpretation of neuron–astrocyte communication is missing.

Transformers, a particular type of artificial intelligence (AI) architecture, have become influential in machine learning (13) and, increasingly, in computational neuroscience (14–20). They are currently the choice model for tasks across many disparate domains, including natural language processing, vision, and speech (21). Interestingly, several recent reports suggested architectural similarities between Transformers and the hippocampus (15, 19) and cerebellum (18), as well as representational similarities with human brain recordings (14, 16, 20). However, unlike more traditional neural networks, such as convolutional networks (22) or Hopfield networks (23), which have a long tradition of biological implementations, Transformers are only at the beginning of their interpretation in terms of known biological processes.

We hypothesize that biological neuron–astrocyte networks can perform the core computations of a Transformer. In support of this hypothesis, we explicitly construct an artificial neuron–astrocyte network whose internal mechanics and outputs approximate those of a Transformer with high probability. The main computational element of our network is the tripartite synapse, the ubiquitous three-factor connection between an astrocyte, a presynaptic neuron, and a postsynaptic neuron (24). We argue that tripartite synapses can perform the role of normalization in the Transformer’s self-attention operation. As such, neuron–astrocyte networks are natural candidates for the biological “hardware” that can be used for computing with Transformers.

The organization of this paper is as follows. We begin with two primers, which introduce the core concepts and notations: one on astrocyte biology and the other one on Transformers. Then, we describe our neuron–astrocyte network in detail and demonstrate the correspondence to Transformers through theory and simulations. We begin by establishing the correspondence for the models with shared weights and then show the general case. For completeness, we also derive a nonastrocytic mechanism for implementing Transformers biologically. Although, ultimately, it should be decided through experiments which of the two mechanisms is closer to biological reality, from the theoretical perspective we argue that astrocytes provide a more natural and parsimonious hypothesis for how Transformers might be implemented in the brain. We conclude with a discussion on the intrinsic timescales of our biological Transformers, as well as potential future work.

## Primer on Astrocyte Biology.

Glial cells are the other major cell type in the brain besides neurons. The exact ratio of glia to neurons is disputed, but it is somewhere between 1:1 and 10:1 (25). The most well-studied type of glial cell is the astrocyte. A defining feature of astrocytes is that a single astrocyte cell forms connections with thousands to millions of nearby synapses (26). For example, a single human astrocyte can cover between 270,000 to 2 million synapses within a single domain (27). Astrocytes are mostly electrically silent, encoding information in the dynamics of intracellular calcium ions ($\;{\text{Ca}}^{2+}$). In most parts of the brain, neurons and astrocytes are closely intertwined. For example, in the hippocampus as many as 60% of all axon–dendrite synapses are wrapped by astrocyte cell membranes called processes (28). In the cerebellum, the number is even higher. This three-way arrangement (presynaptic axon, postsynaptic dendrite, astrocytes process) is so common that it has been given a name: the tripartite synapse (24).

Astrocyte processes contain receptors corresponding to the neurotransmitters released at the synaptic sites they ensheathe. For example, astrocytes in the basal ganglia are sensitive to dopamine, whereas in the cortex astrocytes are sensitive to glutamate (29). Despite being affected by the same presynaptic neurotransmitters, postsynaptic neurons and astrocytes respond very differently: Neurons primarily encode information using action potentials, but astrocytes encode information via elevations in free intercellular calcium. Importantly, neuron-to-astrocyte signaling can trigger a response in the opposite astrocyte-to-neuron direction thus establishing a feedback loop between neural cells and astrocytes. Astrocytes can either depress or facilitate synapses, depending on the situation (30). For example, astrocytes in the hypothalamus have been observed to multiplicatively scale the excitatory synapses they ensheathe by the same common factor (31).

Interestingly, there is also extensive astrocyte-to-astrocyte communication in the brain. Astrocytes form large-scale networks with one another (26). These networks are spatially tiled, with regular intercellular spacing of a few tens of micrometers (32). Unlike neurons, which communicate primarily with spikes, astrocytes communicate via calcium waves that propagate between their cell bodies, processes, and endfeet (33). These waves have speeds of a few tens of micrometers per second. It is thought that these waves could be used to synchronize neural populations and coordinate important neural processes (34).

Among this plethora of biological phenomena, the following four points will be important for our mathematical model:

- Most synapses in the brain are tripartite (presynaptic neuron, postsynaptic neuron, astrocyte process).
- There is a feedback loop between astrocyte processes and synapses. Astrocyte processes respond to presynaptic neural activity with an elevation in intracellular calcium ions ($\;{\text{Ca}}^{2+}$) and, in turn, release gliotransmitters which modulate synapses. This modulation can be either facilitating or depressing.
- The neuron → astrocyte signaling pathway is plastic.
- Nearby astrocyte processes can spatially average their $\;{\text{Ca}}^{2+}$ levels.

Next, we introduce Transformers from the AI perspective, before proposing their biological implementation with astrocytes.

## Primer on Transformers.

Transformers (13) are a popular neural architecture used in many of the recent innovations in AI including Foundation Models (35), Generative Pre-trained Transformer-3 (GPT3) (36), Chat Generative Pre-trained Transformer (ChatGPT) (37), etc. Originally developed for natural language processing tasks, Transformers are taking over the leader boards in other domains too, including vision (38), speech, and audio processing (21). Initially, Transformers were developed as a means to overcome the shortcomings of recurrent neural networks (13). A major difference between these two architectures is as follows: while recurrent neural network process inputs one at a time, Transformers have direct access to all past inputs. Through their self-attention mechanism (described in detail shortly), Transformers can learn long-range dependencies between words in a sentence without having to recurrently maintain a hidden state over long time intervals. Among other computational benefits, this allows for more efficient parallelization during the training process and avoids the vanishing/exploding gradient problem (39–41). In the vision domain, Transformers have also achieved state-of-the-art results (38) surpassing convolutional neural networks. While the latter use hard-coded inductive biases enabling them to learn local correlations between pixels in the images plane, Transformers form long-range learnable dependencies in the image plane right away starting from the early layers of processing (42).

Although recurrent and convolutional neural networks admit straightforward biological interpretations, Transformers presently do not. The reason has to do with the Transformer’s self-attention mechanism. In particular, the so-called self-attention matrix is computed by a) calculating all pairwise dot products between “tokens” (e.g., words in a sentence, patches in an image, etc), b) exponentiating these dot product terms, and then c) normalizing the rows of this matrix to sum to one. These operations are fundamentally nonlocal in time and space, which make them difficult to interpret in biological terms. Later on, we will show how astrocyte biology offers a biologically plausible solution to this dilemma.

Transformers are typically a composition of many Transformer “blocks.” A typical Transformer block uses four basic operations: self-attention, feed-forward neural network, layer normalization, and skip connections. These operations are arranged in a certain way so that the entire block can learn relationships between the tokens, which represent the data. More formally, consider a sequence of N token embeddings. Each token can correspond to a word (or a part of the word) if the Transformer is used in the language domain or a patch of an image in the vision domain. Each embedding is of dimension d. The tokens are streamed into the network one by one (online setting), and the time of the token’s presentation is denoted by t. The $t^{\mathrm{th}}$ embedding is given by a vector $x_{t}\in R^{d}$. Going forward, it will be helpful to collect these tokens into a single matrix, X:

[1] $$\begin{array}{c} X\equiv \;\left[\begin{array}{cccc} | & | & \; & |\\ x_{1} & x_{2} & \cdots & x_{N}\\ | & | & \; & | \end{array}\right]\;\in R^{d\times N}. \end{array}$$

In the Transformer block, each token is converted to a key, query, and value vector via a corresponding linear transformation: $W_{K},W_{Q}\in R^{D\times d}$ and $W_{V}\in R^{d\times d}$. Here, D is the internal size of the attention operation. These transformations are optimized during training. The key, value, and query vectors are then collected into matrices, similarly to Eq. **1**:

[2] $$\begin{array}{c} \begin{array}{c} k_{t}=W_{K}x_{t}\\ v_{t}=W_{V}x_{t}\\ q_{t}=W_{Q}x_{t} \end{array}\to \begin{array}{c} K=W_{K}X\\ V=W_{V}X,\\ Q=W_{Q}X \end{array} \end{array}$$

After computing the key, value, and query matrices, the next major step in a Transformer is the self-attention operation, which allows the tokens to exchange information with each other. The self-attention matrix, SelfAttn(X), is an N×N matrix which contains information about all the pairwise interactions between tokens. At the core of the self-attention mechanism is the softmax function. Recall that the softmax function exponentiates the elements of a vector and then divides each element by the sum of these exponentials. Denoting column t of the self-attention matrix by attn(t), we have that

$$\begin{array}{c} \;\text{attn}\left(t\right)=\sum_{i=1}^{N}\alpha_{i}\left(t\right)v_{i}\text{ with }\alpha_{i}\left(t\right)=\frac{e^{k_{i}^{T}q_{t}}}{\sum_{j=1}^{N}e^{k_{j}^{T}q_{t}}}. \end{array}$$

**Fig. 1.**
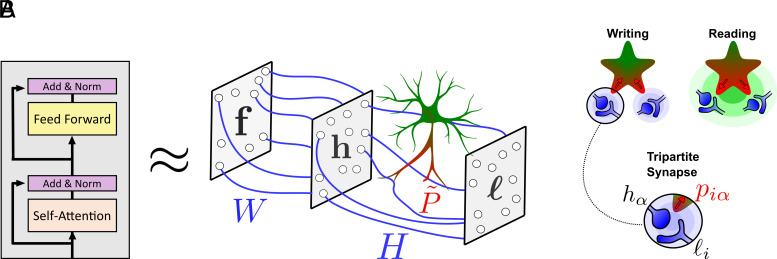
(*A*) A high-level overview of the proposed neuron–astrocyte network. The Transformer block is approximated by a feed-forward network with an astrocyte unit that ensheaths the synapses between the hidden and last layers (matrix H). Data are constantly streamed into the network. (*B*) During the writing phase the neuron-to-neuron weights are updated using Hebbian learning rule and the neuron-to-astrocyte weights are updated using a presynaptic plasticity rule. During the reading phase, the data are forwarded through the network, and the astrocyte modulates the synaptic weights H.

Due to the softmax normalization, each column of the self-attention matrix can be interpreted as a convex combination of the value vectors. Given this definition as well as Eq. **2**, we can write the self-attention matrix compactly as:

[3] $$\begin{array}{c} \;\text{SelfAttn}\left(X\right)=V\;\text{softmax}\left(K^{T}Q\right), \end{array}$$

where here the softmax normalization is computed along the *columns* of $K^{T}Q$. The output of this self-attention operation is then passed along to a LayerNorm operation and a feed-forward neural network (FFN) that both act separately on each token (each column of its input), see Fig. 1. Recall that a LayerNorm scales each element of a vector by the mean and variance of all elements in the vector (43) and can be implemented in a biologically plausible manner (44). Without loss of generality, a single-headed attention Transformer is studied. In this case, the output of the full Transformer block may be written as a two-step process:

[4] $$\begin{array}{c} \begin{array}{cc} Y & =\;\text{LayerNorm}(\;\text{SelfAttn}(X)+X)\\ \;\text{Transformer}(X) & =\;\text{LayerNorm}(\;\text{FFN}(Y)+Y), \end{array} \end{array}$$

where FFN refers to a feedfoward network, applied to each token (i.e., each column of Y) separately and identically.

## Biological Implementation of a Transformer Block

In order to gain theoretical insight into Transformers, it is common to tie the weights (45, 46). This tying can be within a single Transformer block, between blocks, or both. In this section, we will tie the weights within a single block but not between blocks. We will relax this weight sharing constraint in the later sections. In particular, we tie $W_{Q},W_{K},W_{V}$ as follows:

[5] $$\begin{array}{c} W_{Q}=W_{K}=W,W_{V}=I, \end{array}$$

for some arbitrary matrix W and the identity matrix, I. In general, we will not require that d=D. We include this constraint now to fully analyze the simplest version of our model that captures the essential elements of our argument. Without loss of generality, we will ignore layer normalization steps for now, returning to them in the section titled “General Case of Untied Weights.”

### Neuron–Astrocyte Network.

A high-level overview of our circuit is shown in Fig. 1. The network consists of a perceptron with an input layer, a hidden layer, and an output layer (Fig. 1*A*). As in many associative memory systems, our network has distinct writing and reading operations (23, 47). In particular, our network alternates between writing and reading *phases* (Fig. 1*B*). The writing phase enables the circuit to store information about all the tokens; the reading phase enables any given token to interact with all the others. Recall that a difficulty with interpreting Transformers as biological circuits is that they require operations which are nonlocal in space and time. Having distinct writing and reading phases allows our network to resolve this temporal nonlocality. As we will see, the spatial nonlocality is resolved through the astrocyte unit.

The d-dimensional inputs are passed to the hidden layer with m units, as well as to the last layer via a skip connection (not shown in Fig. 1). The hidden layer applies a fixed nonlinearity to incoming inputs. The outputs of the hidden layer are passed to the last layer via a linear mapping $H\in R^{d\times m}$. The synapses in the matrix H are triparite synapses, meaning that each of the md synapses is associated with an astrocyte process $p_{i\alpha }$. The Latin indices i,j are used to enumerate neurons in the first and last layers, while the Greek indices α,β are reserved for the hidden neurons. The strength of the synapse between a hidden neuron α and the output neuron i is denoted by $H_{i\alpha }$ and the activity of the astrocyte process that ensheaths this synapse is described by $p_{i\alpha }$. The layers are denoted from left to right as f,h,ℓ (first, hidden, last), respectively. Our network is described by the following equations:

[6] $$\begin{array}{c} \begin{array}{cc} f=x & \in R^{d}\\ h=\phi (Wf) & \in R^{m}\\ \ell =r(H⊙\tilde{P})h+f & \in R^{d}, \end{array} \end{array}$$

The scalar r={0,1} stands for ‘read’ and is zero during the writing phase and unity during the reading phase. The symbol ⊙ denotes the Hadamard product (element-wise multiplication) between two matrices. The matrix $\tilde{P}\in R^{d\times m}$ captures the effect of the astrocyte processes and is defined as follows:

$$\begin{array}{c} {\tilde{P}}_{i\alpha }=\frac{1}{p_{i\alpha }} \end{array}$$

This inverse modulation of synaptic weights by astrocytes has been observed, for example, in studies involving tumour necrosis factor-alpha (TNF-α), wherein astrocytes will upscale synaptic weights in response to low neural activity and downscale weights in response to high neural activity. More generally, many studies have observed that astrocytes can both depress and facilitate synapses, depending on the situation (1, 48–51).

### Neural Activation Function.

The neural activation function ϕ plays a special role in our circuit. In order to match the exponential dot product in the Transformer’s self-attention mechanism, we will require that ϕ be an approximate feature map for the exponential dot product kernel

[7] $$\begin{array}{c} \phi {\left(x\right)}^{T}\phi \left(y\right)\approx e^{x^{T}y}, \end{array}$$

There are many (indeed, infinitely many) activation functions which satisfy this condition. Several biologically plausible options come from the theory of random feature maps (52–54), and we will discuss them in detail later on. For now, we will simply assume that ϕ is chosen so that Eq. **7** is true. More generally, however, one can pick any ϕ such that $\phi {\left(x\right)}^{T}\phi \left(y\right)\geq 0$ to yield a valid self-attention mechanism (55). Nevertheless, only particular choices of ϕ yield the softmax self-attention which is used in most Transformers at scale (13).

### Astrocyte Process Dynamics.

As discussed in the introduction, astrocyte processes are sensitive to presynaptic neural activity. To capture this mathematically, we assume that the astrocyte process $\;{\text{Ca}}^{2+}$ response is linearly proportional to the presynaptic neuron activation $h_{\alpha }$ of neuron α in layer h. The constant of proportionality between the astrocyte process activation and the presynaptic neural activity is denoted as $g_{i\alpha }$. This constant is in general different for every astrocyte process. Upon presentation of an embedded token to the network, astrocyte process $p_{i\alpha }$ initially responds with a local calcium elevation $g_{i\alpha }h_{\alpha }$. This $\;{\text{Ca}}^{2+}$ response is then spatially averaged with the responses of other nearby astrocyte processes so that, after transients, the processes have the same value once a token is presented:

[8] $$\begin{array}{c} p_{i\alpha }=\frac{1}{\mathrm{md}}\sum_{j=1}^{d}\sum_{\beta =1}^{m}g_{j\beta }h_{\beta }=p. \end{array}$$

The neuron-to-astrocyte signaling pathway in our circuit is completely described by Eq. **8**.

### Writing Phase.

During the writing phase, r is set to zero. Biologically, this condition could correspond to some global neuromodulator being released into the local environment, for example, acetylcholine, as suggested in refs. 17 and 56. Plugging r=0, Eq. **6** becomes

[9] $$\begin{array}{c} \begin{array}{c} f_{t}=x_{t}\\ h_{t}=\phi \left(Wf_{t}\right)=\phi \left(k_{t}\right)\\ \ell_{t}=f_{t}=v_{t}, \end{array} \end{array}$$

where we have substituted in the definitions of the key, query, and value vectors given by Eq. **2**, as well as the temporary weight-tying assumption given by Eq. **5**. As the embedded tokens are passed into Eq. **9** sequentially, the weight matrix H is updated via Hebbian plasticity with a learning rate of $\frac{1}{m}$. Upon presentation of token t, the matrix H is

$$\begin{array}{c} H_{t}=H_{t-1}+\frac{1}{m}\ell_{t}h_{t}^{T}\Rightarrow H=\frac{1}{m}V\phi^{T}\left(K\right), \end{array}$$

where we have assumed that H is initially the zero matrix and substituted in the equalities in Eq. **9**. At the same time that the neuron-to-neuron weights are updated via Hebbian plasticity, the neuron-to-astrocyte weights are updated via presynaptic plasticity. Upon presentation of token t, these weights are

$$\begin{array}{c} g_{t,i\alpha }=g_{t-1,i\alpha }+\phi {\left(Wx_{t}\right)}_{\alpha }\Rightarrow g_{i\alpha }=\sum_{j=1}^{N}\phi {\left(k_{j}\right)}_{\alpha }. \end{array}$$

Note that as a consequence of the presynaptic plasticity, the weight $g_{i\alpha }$ does not depend on the index i. Therefore, we will only refer to the vector $g\in R^{m}$, which—through the presynaptic plasticity—is simply the sum over all token presentations of the hidden layer neural activations:

$$\begin{array}{c} g=\sum_{j=1}^{N}\phi \left(k_{j}\right). \end{array}$$

### Reading Phase.

During the reading phase, the read gate is set to r=1 in Eq. **6**, and the inputs are forwarded through the network. The astrocyte process activation value p, which according to Eq. **8** does not depend on indices i and α, is given by

[10] $$\begin{array}{c} \begin{array}{c} p=\frac{d}{\mathrm{md}}g^{T}h=\frac{1}{m}\sum_{j=1}^{N}\phi {\left(k_{j}\right)}^{T}\phi \left(q_{t}\right). \end{array} \end{array}$$

To obtain the last equality, we have used $h_{t}=\phi \left(Wx_{t}\right)=\phi \left(q_{t}\right)$. Plugging in all the steps of Eq. **6**, we see that the last layer has the following output

[11] $$\begin{array}{cc} \ell_{t} & =\frac{1}{p}H\phi \left(q_{t}\right)+x_{t}=\frac{V\phi^{T}\left(K\right)\phi \left(q_{t}\right)}{\phi {\left(q_{t}\right)}^{T}\sum_{j=1}^{N}\phi \left(k_{j}\right)}+x_{t}\\ & \approx \sum_{i=1}^{N}\frac{e^{k_{i}^{T}q_{t}}}{\sum_{j=1}^{N}e^{k_{j}^{T}q_{t}}}v_{i}+x_{t}\\ & =\;\text{attn}\left(t\right)+x_{t}, \end{array}$$

where we have used the assumption that ϕ is an approximate feature map for the exponential dot product, given by Eq. **7**. If we compute $\ell_{t}$ for every token $x_{t}$ and stack the results column-wise into a matrix L, we can conclude that the output of our neuron–astrocyte circuit is approximately the output of the Transformer’s self-attention, plus the necessary residual connection:

[12] $$\begin{array}{c} \begin{array}{c} L\approx \;\text{SelfAttn}(X)+X. \end{array} \end{array}$$

### Random Feature Activations.

As mentioned above, in order to approximate the softmax attention, we require that ϕ is a feature map for the exponential dot product. This is the idea behind linear Transformer architectures (55) such as Performers (53) and Random Feature Attention (54). We will now discuss two biologically plausible options for such a feature map. The first relies on a well-known result in kernel approximation theory (52), which is that the radial basis function (RBF) kernel can, with high probability, be approximated very well using random projections and cosines

[13] $$\begin{array}{c} \phi \left(x\right)=\sqrt{\frac{2}{m}}\;\text{exp}(\frac{{\left||x|\right|}^{2}}{2})\;\text{cos}\left(\Pi x+b\right), \end{array}$$

where the elements of $\Pi \in R^{m\times D}$ are drawn from a standard normal distribution, and the elements of $b\in R^{m}$ are drawn from the uniform distribution on [0,2π]. A related but different random feature map was introduced in the context of Performers (53). There it was shown that instead of cosines, one can just as well use exponential functions

[14] $$\begin{array}{c} \phi \left(x\right)=\sqrt{\frac{1}{m}}\;\text{exp}(\frac{-{\left||x|\right|}^{2}}{2})\;\text{exp}\left(\Pi x\right), \end{array}$$

Note that due to the softmax normalization, any constant prefactors in Eq. **13** can be ignored (since they cancel in the numerator and denominator). If we assume an additional spherical normalization step before the random projection layer, so that all arguments to ϕ have constant norm, then the above activation functions may be written more plainly as

$$\begin{array}{c} \phi (x)=\;\text{cos}(\Pi x+b)\;\text{and }\phi (x)=\;\text{exp}(\Pi x). \end{array}$$

Cosine tuning curves appear ubiquitously in neuroscience, across many different organisms (e.g., crickets, cats, rhesus monkeys) and many different brain areas (e.g., cerebellum, motor cortex, and hippocampus) (57, 58). The function exp(·) is monotonic and positive, making it easy to implement from a biological perspective. For the exponential random feature function, the term $\;\text{exp}(\frac{-{\left||x|\right|}^{2}}{2})$ may be interpreted as a homeostatic mechanism to ensure that firing rates do not become too large. We stress that while the aforementioned random feature maps are sufficient for approximating the softmax self-attention mechanism, there are infinitely many other activation functions that lead to valid (though potentially nonsoftmax) self-attention matrices.

## General Case of Untied Weights

In this section, we relax the weight tying condition and generalize our construction to the case when D≠d. While in the previous sections r acted as a gatekeeper for the weight matrix H, we will now *also* have r act as a gatekeeper for a few other weight matrices. Using the same variable names, consider the following neuron–astrocyte forward equations:

[15] $$\begin{array}{c} \begin{array}{cc} f=x & \in R^{d}\\ h=\phi [\left(1-r\right)W_{K}f+rW_{Q}f] & \in R^{m}\\ \ell =r\left(H⊙\tilde{P}\right)h+\left(1-r\right)W_{V}f+rf & \in R^{d}, \end{array} \end{array}$$

**Fig. 2.**
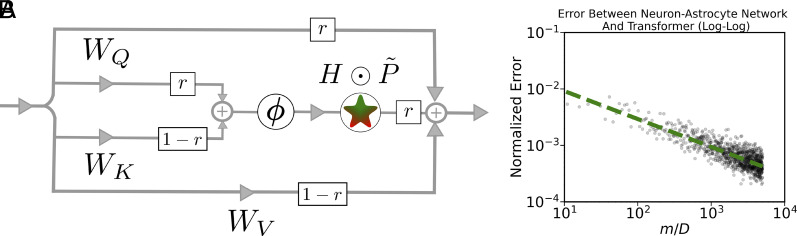
(*A*) Circuit diagram of the full neuron–astrocyte model Eq. **15**, which implements a general (i.e., untied) Transformer block. (*B*) Error vs number of hidden units (m) in our network. As m increases, the difference between the output of the neuron–astrocyte circuit and the AI Transformer block decreases.

When r=0, we recover the writing phase of Eq. **9**; when r=1, we recover the reading phase equations of Eq. **11**. When we impose the weight tying constraint of $W_{K}=W_{Q}=W$ and $W_{V}=I$, we recover the original equations of Eq. **6**. Eq. **15** describes the neuron–astrocyte implementation of the general Transformer block without the weight sharing constraint imposed. The circuit diagram corresponding to Eq. **15** can be seen in Fig. 2*A*.

## Numerical Validation

**Fig. 3.**
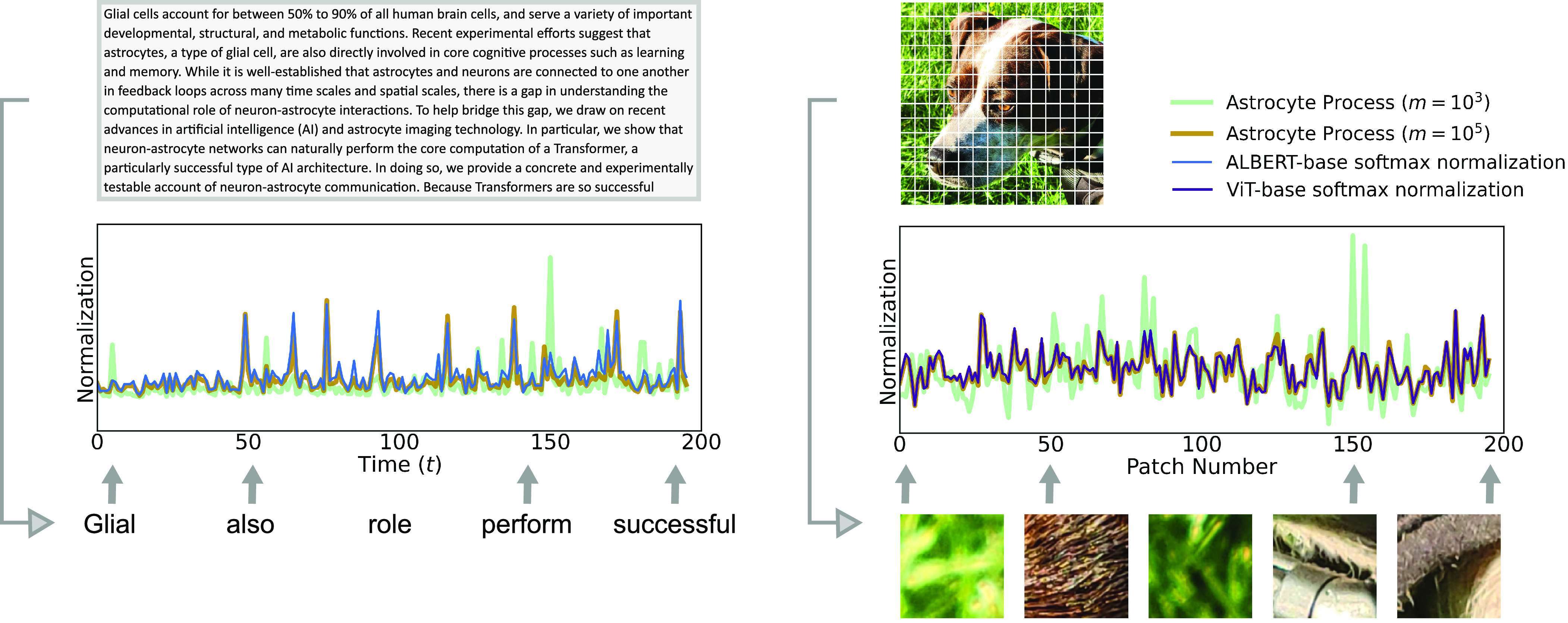
(*Left*) Astrocyte traces for $m=10^{3}$ and $m=10^{5}$ compared against the “exact” softmax normalization terms of the ALBERT-base model. The tokens used were the first 200 words of the abstract of this paper. See “Numerical Validation” for details. (*Right*) Similar plot as in left side, but for a Vision Transformer. Instead of using embedded words as tokens, the model uses patches from an image.

The results derived above have also been checked numerically. In Fig. 2*B*, one can see the error between the proposed neuron–astrocyte network and the actual AI Transformer block as a function of the ratio of the width of the hidden layer to the size of the token embedding. As expected from the theoretical analysis, the error between the two networks rapidly decreases as the hidden layer becomes wider. In practice, as the width of the hidden layer becomes 5 to 10 times the embedding dimension, the two networks produce very similar outputs. In Fig. 3*A*, we use the parameters of the ALBERT-base (59, 60) Transformer to generate a corresponding neuron–astrocyte model. In particular, we extracted the word embedding matrix, the encoder matrix, and the $W_{Q}$, $W_{K}$, $W_{V}$ matrices from the first block of ALBERT-base. We then embedded and encoded the first 200 words of the abstract of this paper. We plugged these weights into two neuron–astrocyte networks Eq. **15**—one with $m=10^{3}$ hidden neurons and one with $m=10^{5}$ hidden neurons—and passed the tokens through the network. We extracted the astrocyte responses during the reading phase and plotted these along with the actual softmax normalization terms in ALBERT-base model. In Fig. 3*B*, we performed a similar “weight transfer” from a Vision Transformer model that was pretrained on ImageNet-21K (61, 62). In this case, the tokens were patches of an image, instead of words in a sentence. As expected from the theoretical derivation, for sufficiently large number of hidden units, neuron–astrocyte networks accurately describe computation performed by the Transformer models. The code to reproduce Fig. 3 is available in the following GitHub repository: https://github.com/kozleo/neuron-astrocyte-transformer.

## Do We Need Astrocytes?

Although we are interested in addressing the scientific problem of how astrocytes participate in behavior, a natural question when positing any new brain mechanism is as follows: “Can the same behavior be achieved without this mechanism?” This section demonstrates that a Transformer circuit can *also* be constructed using neurons and bipartite synapses, together with a specialized divisive normalization achieved via shunting inhibition. The circuit is similar to Eq. **6**:

[16] $$\begin{array}{c} \begin{array}{cc} f=x & \in R^{d}\\ h=\phi (Wf) & \in R^{m}\\ R=g^{T}h & \in R\\ \ell =\frac{r}{R}Hh+f & \in R^{d}. \end{array} \end{array}$$

The only difference between Eqs. **16** and **6** is the addition of a new element, R, and the removal of the astrocyte processes. Here, R is an inhibitory neuron that divisively normalizes feed-forward inputs into layer ℓ. However, it does not inhibit all feedfoward inputs equally. Despite both h and f being feed-forward inputs to layer ℓ, the divisive inhibition is only implemented on the inputs coming from layer h. This can happen, for example, if the feed-forward synaptic inputs coming from layer h arrive at the dendritic tree close to where inhibitory inputs from neuron R shunt current flow, while the feed-forward inputs coming from layer f synapse far away from the shunting (63). Leaving the reading and writing phases untouched, circuit Eq. **16** implements the same forward pass as Eq. **6**.

While the proposed nonastrocytic circuit can, in theory, also implement a Transformer forward pass, it should be noted that there exists a controversy about the capability of shunting inhibition to implement divisive normalization (63, 64). Thus, the biologically plausibility of this circuit is questionable. Additionally—as we will discuss in the next section—the comparatively slower timescale of astrocytes provides a natural memory buffer when, e.g., accumulating and storing words in a sentence. Finally, it is possible that there are *many* ways to implement Transformers biologically, each with relative pros and cons. Different brain areas may implement Transformer-like computation using different circuitries. It is ultimately an experimental question to validate these theoretical hypothesis.

## Timescales

One aspect of our model which we have yet to discuss is its timescale. Our circuit operates in two distinct phases: a reading phase and a writing phase. The reading phase does not involve any plasticity, so the only relevant timescale to compute is how long it takes to traverse the neuron–astrocyte-synapse pathway. Recent data indicate that astrocytes can sense and respond to neural activity on the order of a few hundreds of milliseconds (9, 65). The speed of the writing phase is limited by the speed of plasticity. There are two types of plasticity used in our model during the writing phase: 1) Hebbian plasticity between neurons and 2) presynaptic plasticity between neurons and astrocytic processes. In the case of neuron–neuron plasticity, there are experimental studies reporting a vast range of the relevant timescales. These include Hebbian plasticity (66–68), behavioral timescale plasticity (69–71), etc. The induction timescales for these plasticity mechanisms range from hundreds of milliseconds (70) to tens of minutes (67). In the case of STDP computational modeling studies, it is typically assumed that synaptic weights are adjusted instantaneously, by an amount proportional to the timing difference between pre-post synaptic spikes (72, 73). The neuron–astrocyte plasticity timescale is harder to establish, due to limitations in calcium recording technology. While fast calcium transients in astrocyte processes have been recently recorded (9), and neuron–astrocyte plasticity has been experimentally observed (74), fast (e.g., <1 s) neuron–astrocyte plasticity has not been observed yet, possibly due to limitations of the calcium imaging technology.

## Discussion

Here, we have built a computational neuron–astrocyte model which is functionally equivalent to an important AI architecture: the Transformer. This model serves a dual purpose. The first purpose is to provide a concrete, normative, computational account of how the communication between astrocytes and neurons subserves brain function. The second purpose is to provide a biologically plausible account of how Transformers might be implemented in the brain. While the feedback loop between neurons and astrocytes is well studied from an experimental perspective, there is comparatively little work studying it from the computational perspective (7). Astrocyte modeling studies tend to focus on either the biophysics of neuron-astrocyte or astrocyte signaling (75, 76) or the emergent computational properties of detailed neuron-astrocyte models (77–79). Fewer studies have focused on simpler, normative models of neuron–astrocyte networks (51, 80, 81).

An important feature of our model is that it is flexible enough to approximate any Transformer. In other words, we do not only show how to model a particular Transformer (i.e., one with weights that have already been trained for some specific task)—rather, we show how to approximate all *possible* Transformers using neurons and astrocytes. Given the demonstrated power and flexibility of Transformers, this generality can help to explain why astrocytes are so prevalent across disparate brain areas and species. Our model has several immediate implications. First, as calcium imaging technologies improve, it will become increasingly feasible to explicitly compare artificial representations in AI networks to representations in biological astrocyte networks—as is already done when comparing AI networks to biological neural networks (16, 22, 82). Given that astrocyte activity is thought to be tightly coupled to fMRI responses (83), natural language processing contexts such as (16) and (84) are already a promising place to look for astrocytic contributions to brain function. Additionally, we propose that our hypothesis could be refuted through studies involving targeted astrocyte manipulations. The brain’s sensitivity to normal astrocyte function levels is evident. For instance, prior experimental studies have demonstrated that hippocampal astrocyte activation positively influences memory-related behaviors (85), whereas striatal astrocyte activation impairs attention (86). To challenge our hypothesis, we could train both a Transformer model and an animal subject to perform the same hippocampal-based memory task, such as one requiring path integration. Based on previous research, we anticipate a strong correlation between Transformer and hippocampal activations (87). If we could then selectively silence or modify hippocampal astrocytes in the animal subject and demonstrate that the representational similarity to the Transformer model remains unaffected, our hypothesis would be undermined. The main constraint of this approach lies in the present challenge of selectively inactivating astrocytes in a controlled and reversible fashion (1). Nevertheless, we anticipate that advancements in the field of astrocyte biology will eventually overcome these limitations.

Despite the exciting potential links between Transformers and the brain, it is worth noting that humans learn quite differently from Transformers. Transformers are extremely data-hungry, and consequently, training them requires a massive amount of energy (88). By contrast, the human brain runs on a smaller energy budget than a common laptop and does not require internet-scale training datasets to learn a language (89). In view of this fact, it may be more appropriate to view training a large Transformer as analogous to learning over evolutionary timescales, rather than the lifetime of a single individual (90).

Finally, a major roadblock in accepting Transformers as models of natural language processing (or, more generally, sequential processing) in the brain is that they require a memory buffer to store the tokens as they are presented. This is because the self-attention matrix is computed over all the tokens. Our paper proposes that neuron–astrocyte networks can perform this buffering naturally through spatial and temporal integration. Finally, and more speculatively, since astrocytes are implicated in many brain disorders and diseases, our work suggests that causal manipulations on Transformers can be used as a way to generate putative hypotheses for how astrocyte function goes astray in brain disorders and diseases (91, 92).

## Data Availability

There are no data underlying this work. The code used in this work is available at GitHub repository (https://github.com/kozleo/neuron-astrocyte-transformer) (93).
